# Recurrent pyogenic infections caused by a novel Gln1420* mutation in the C3 gene

**DOI:** 10.3389/fped.2022.1017195

**Published:** 2022-10-05

**Authors:** Pedro Simão Coelho, Catarina Gouveia, Marta Valente Pinto, Conceição Neves, Ana Isabel Cordeiro, João Farela Neves

**Affiliations:** ^1^ Primary Immunodeficiencies Unit, Hospital Dona Estefânia, CHULC-EPE, Lisbon, Portugal; ^2^ Infectious Disease Unit, Hospital Dona Estefânia, CHULC-EPE, Lisbon, Portugal; ^3^ Comprehensive Health Research Centre (CHRC), NOVA Medical School, NOVA University of Lisbon, Lisbon, Portugal; ^4^ Chronic Diseases Research Center (CEDOC), NOVA Medical School, NOVA University of Lisbon, Lisbon, Portugal

**Keywords:** primary immunodeficiency, complement deficiency, C3 deficiency, C3 gene mutation, recurrent infections

## Abstract

C3 is a crucial protein of the complement system. Congenital C3 deficiency is extremely rare and manifests through recurrent, severe infections and should always be considered as a differential diagnosis of recurrent pyogenic infections. We report a case of a patient with a novel C3 gene mutation, responsible for complete C3 deficiency with impaired complement system activation and recurrent infections.

## Introduction

Due to anatomical particularities and to the immaturity of the immune system, recurrent infections are very frequent in children. This can lead to a delay in the identification of the patients with congenital defects of immunity. Among children with recurrent infections, those presenting with pyogenic infections caused by encapsulated bacteria, such as *Streptococcus pneumoniae*, *Neisseria meningitidis* or *Haemophilus influenzae*, should always be screened for an underlying primary immunodeficiency (PID) (1). PIDs are a heterogeneous group of monogenic disorders that affect the development and/or function of both innate and adaptive immune system. PIDs can present as frequent, recurrent, or persistent infections, but also as early-onset and/or severe autoimmunity and inflammation (1).

The complement system is part of the immune system and participates not only in host defense against infections but also in the clearance of debris and in the maintenance of a homeostatic inflammatory status (2). In host defense, it is one of the first components engaged by pathogens and its two major functions are mediated by opsonization and membrane perturbation (3). C3 cleavage is the converging point of all complement pathways and, therefore, it is a central step of the complement system (4). Immune complexes are a potent activator of the complement system, and in normal circumstances, are maintained in a soluble state by C3, preventing them to act as a stimulus for inflammation. When C3 is compromised, immune complexes may deposit on endothelial cells and trigger an inflammatory response (3).

Inherited C3 deficiency is an extremely rare primary immunodeficiency, with, until recently, approximately 40 cases described. Its main clinical features are severe recurrent infections mainly caused by encapsulated bacteria, as well as autoimmunity and diseases caused by immune complexes deposition, such as membranous glomerulonephritis (5, 6).

We report a case of a patient with a novel C3 gene mutation responsible for complete C3 deficiency presenting with severe and recurrent bacterial infections.

## Case report

**Figure 1:**
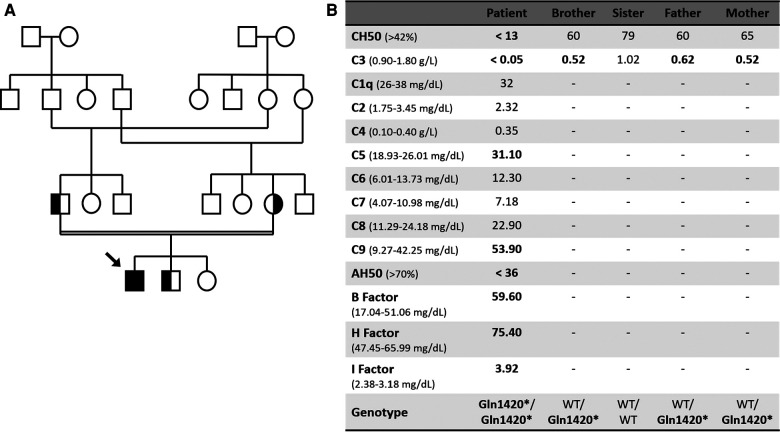
(**A**) Family tree. (**B**) Complement laboratory evaluation. WT, Wild type.

A 4-year-old boy was admitted to the hospital with serogroup B *Neisseria meningitidis* septic shock and meningitis requiring fluid resuscitation and ceftriaxone. His family history was remarkable for parental consanguinity (parents were second degree cousins, Figure 1A) and he had a personal history of recurrent pyogenic infections since birth, with previous hospital admissions caused by orbital cellulitis and *Streptococcus pneumoniae* lobar pneumonia. He also presented recurrent acute otitis media and tonsillitis.

Diagnostic workup revealed undetectable classical complement pathway activity (CH50) and C3 levels (Figure 1B). Further study demonstrated a reduced alternative pathway activity with increased complement B, H and I factors, and raised C5 and C9 levels, probably related to an underlying inflammatory status (Figure 1B). Immunoglobulin G, A and M were within normal range. There was no clinical or laboratory evidence of organ damage after resolution of the infectious disease. The patient was suspected to have a C3 deficiency and was promptly vaccinated against meningococcus and pneumococcus and, because he had frequent and severe infections, antibiotic prophylaxis with amoxicillin was commenced with good adherence and no more hospital admissions. Genetic testing was performed using Illumina next-generation sequencing for a panel of genes involved in complement deficiencies, and revealed a novel homozygotic nonsense mutation in the C3 gene (p.Gln1420*)) that leads to a stop codon, thus being predicted to be deleterious and pathogenic. The mutation was confirmed by Sanger sequencing. The patient's family was screened and both parents and his 3-year-old brother were shown to be healthy carriers of the mutation (Figures 1A,B). The patient is being monitored quarterly and has been presenting good evolution, free from severe infections. Genetic counselling was offered to the family.

## Discussion

C3 is a major component of the complement system. It is the converging point of all three pathways and is responsible for full activation of the complement system. It is also involved in a variety of homeostatic processes, such as tissue regeneration and tumor cell progression control (6, 7). It derives from a precursor molecule with 1663 amino acids encompassed by 41 exons located on chromosome 19. C3 mRNA is translated into pro-C3 and cleaved into β chain (exons 1–16) and α chain (exons 16–41), which are subsequently linked by disulfide bonds to make the mature C3 molecule (8).

The C3 is cleaved by both classical (C4bC2a) and alternative (C3bBb) pathway convertases, resulting in C3a and C3b. C3a is an anaphylatoxin responsible for degranulation of mast cells and basophils, leading to inflammatory reactions (3, 9). C3b is the major opsonin of the complement system and promotes adherence between opsonized microbes and immune cells. Through iC3b, a product of C3b degradation, enhances complement mediated phagocytosis and acts as a natural adjuvant for antibody production by reducing B cell receptor stimulation threshold (7, 10).

A dysregulation of the complement system may be involved in a decrease in functional activity that leads to increased susceptibility to bacterial infections, particularly pneumococcal for early and meningococcal for late pathway, autoimmunity and undesirable tissue damage (3). Complement deficiencies are considered rare, but they are estimated to be responsible for between 1% and 10% of all PIDs (9). Most congenital complement deficiencies display an autosomal recessive inheritance and heterozygous carriers usually remain asymptomatic, with defects being identified through medical history and family analysis (9). In most cases, the onset of infections begins in childhood but the undervaluation of this type of disease may reflects a lack of awareness among practitioners (6). Lack of C3, besides recurrent encapsulated pyogenic bacteria infections, also leads to an impaired inflammatory response and decreases the patient's ability to clear immune complexes, resulting in renal, pulmonary and vascular damage and might also promote the development of antibodies to self-antigens (3, 9), which did not happen in our patient.

The European Society of Immunodeficiencies has issued recommendations regarding management of complement deficiencies, with emphasis on vaccination, namely on conjugated vaccines against pneumococcus, *Haemophilus influenzae* and *Neisseria meningitidis*, as one of the most important tools to prevent severe infections (5, 11).

In the case of C3 deficiency, due to the increased susceptibility to invasive pneumococcal infections and recurrent pyogenic infections, some authors recommend that in addition to vaccination it may be important to evaluate pneumococcal antibody levels, since patients with low level responses should receive booster vaccinations (5).

Regarding antibiotics, there is no formal recommendation and should therefore be evaluated for every patient, based on an individualized risk stratification. Most patients with complement deficiencies may be managed with an as-needed plan, with prompt use within the first signs of infection. Antibiotic prophylaxis is controversial, as the benefits should be balanced against the risks, particularly the development of resistance to antibiotics. When used, it may be penicillin or macrolide-based, as it should target encapsulated bacteria (5, 12). The authors propose that it should only be used in patients with recurrent infections despite appropriate vaccination, namely in those with difficult access to healthcare services, as this may delay medical assistance and worsen their prognosis.

In summary, we report the first Portuguese patient with complete C3 deficiency caused by a novel C3 mutation that was diagnosed after a severe meningococcal infection on a patient with recurrent pyogenic infections and with a consanguineous family.

## Data Availability

The raw data supporting the conclusions of this article will be made available by the authors, without undue reservation.
